# Grazing and topography control nutrient pools in low Arctic soils of Southwest Greenland

**DOI:** 10.1111/ejss.13278

**Published:** 2022-07-24

**Authors:** Maud A. J. van Soest, N. John Anderson, Roland Bol, Liz R. Dixon, Philip M. Haygarth

**Affiliations:** ^1^ Geography & Environment Loughborough University Loughborough UK; ^2^ Department of Ecology and Environmental Science Umeå University Umeå Sweden; ^3^ Institute of Bio and Geosciences, Agrosphere (IBG‐3), Forschungszentrum Jülich GmbH Jülich Germany; ^4^ School of Natural Sciences, Environment Centre Wales Bangor University Bangor UK; ^5^ Rothamsted Research, North Wyke Okehampton UK; ^6^ Lancaster Environment Centre Lancaster University Lancaster UK; ^7^ Present address: Centre for Ecology and Hydrology, Environment Centre Wales Bangor UK

**Keywords:** carbon, muskox, nitrogen, nutrients, phosphorus

## Abstract

Soil nutrient pools in the dry low Arctic are likely to be released under climatic change and this bioavailability has the potential to increase both terrestrial and aquatic productions. As well as the direct effect of warming, external disturbances such as nutrient deposition and grazing can also drive ecosystem change. This study in the low Arctic Kangerlussuaq area of southwest Greenland compared soil nutrient pools in terms of both topographic position on a catena and by soil depth in two small catchments with contrasting muskox abundance. We tested the hypotheses that there were differences between soil carbon (C), nitrogen (N) and phosphorus (P) across a soil catena (ridge ‐ slope ‐ valley) and by soil depth (litter ‐ 0–5 cm ‐ 25–30 cm) for the two sites (SS17b, muskox present, versus ‐ SS85, no muskox). Total C and N concentrations of soils were on average lower at SS17b compared to SS85. Moreover, the soil N concentration increased downslope in the catena with higher amounts in the valleys compared to the slopes and ridges. Soil P concentration (0.70 g P kg^−1^) was similar between catchments; however, litter P content was substantially different. The difference in soil nutrients between the two catchments was most likely due to the presence of muskox at SS17b, and hence grazing associated processes (defecation, altered microbiology and nutrient cycling). This study emphasises the heterogeneity of arctic landscapes and need for ecosystem specific research.

**Highlights:**

- Soil nutrient pools in two low‐arctic catchments in Greenland were compared.
- Grazing and dung inputs by muskox affect soil nutrient pools in Greenland.
- Soil P stores in Kangerlussuaq are similar to intensively managed farmland in Europe.
- The heterogeneity of arctic landscapes and need for ecosystem‐specific research are emphasised.

## 1 INTRODUCTION

Arctic soils store substantial amounts of organic matter, predominantly sequestered in the permafrost matrix, which may become bioavailable in a warmer future (Schuur et al., 2015). However, the size of this pool and storage is poorly understood (Hugelius et al., 2013) and the biogeochemical response to permafrost degradation can be site specific (Tank et al., 2020). Deepening of the active layer in arctic soils is likely altering soil microbiology and nutrient dynamics resulting in mobilisation of nitrogen (N) and phosphorus (P) (Hobbie et al., 2002) as well as increasing CO_2_ release to the atmosphere (Hugelius et al., 2013). Moreover, geomorphological and hydrological changes are expected under warmer temperatures, particularly through increasing connectivity between groundwater and surface water (Bring et al., 2016). These changes have substantial implications for the functioning of terrestrial and aquatic ecosystems via the release of nutrient stores and associated distribution along surface and subsurface hydrological pathways (Vonk et al., 2019; Wookey et al., 2009).

While much of the focus to date has been on pools of organic carbon (C) in arctic soils, the N and P content is also ecologically important (Aanderud et al., 2018). Carbon, N and P compose the core of elemental cycling and their stoichiometry is believed to be generally stable in undisturbed ecosystems (Redfield, 1958). However, climate change is causing a decoupling of C:N:P stoichiometry in both plants and soils (Tipping et al., 2016) and any imbalance in C:N:P stoichiometry could indicate nutrient limitation of ecosystems (Street et al., 2018). These changes can be pronounced at higher latitudes as climate change proceeds rapidly (Serreze & Barry, 2011).

As well as the direct effect of warming, arctic biogeochemistry is also influenced through changes in ecosystem phenology and production associated with longer growing seasons related to climate change. There is a growing debate over arctic greening (Myers‐Smith et al., 2020) and browning (Treharne et al., 2019) related to changing vegetation cover, and the potential consequences for trophic dynamics and soil (Parker et al., 2018; Snyder, 2013; Street et al., 2018). But as well as these autogenic‐driven changes in vegetation and soil processes, external disturbances such as fire (Michaelides et al., 2019), N‐deposition, and grazing can also drive ecosystem change (Jørgenson et al., 2013).

The role of changing ungulate population dynamics and grazing activity has received increased attention in the Arctic recently (e.g. Jørgenson et al., 2013). With a characteristically short growing season, the grazing of photosynthetic tissue can severely restrict shrub productivity and affect ecosystem C storage and allocation (Cahoon et al., 2012). Reindeer (*Rangifer tarandus*) and muskox (*Ovibus moschatus*) are the only large herbivores found on Greenland (Anderson, 2020), with muskox being introduced to west Greenland in 1962 (Raundrup et al., 2012). Post and Forchhammer (2002) compared the population dynamics of muskox and reindeer from both sites of the ice sheet (east vs west Greenland) and found a strong correlation to the North Atlantic Oscillation (NAO) and local winter temperatures. Cahoon et al. (2012) reported the effect of caribou and muskox exclusion on ecosystem CO_2_ exchange near Kangerlussuaq in west Greenland. They found increases in shrub cover, leaf area, ecosystem photosynthesis, and a nearly threefold increase in net C uptake in the absence of herbivores (Cahoon et al., 2012). Shrub coverage was shown to increase in an experimental exclusion of reindeer and muskox (Olofsson et al., 2001), and Jørgenson et al. (2013) showed the capability of muskox to control shrub growth on a landscape scale without experimental exclusion.

As well as the effects of large herbivores such as reindeer and muskox, outbreaks of the larvae of the *Eurois occulta* moth have been documented in relation to widespread defoliation of dwarf shrub tundra (Avery & Post, 2013). Geese have been shown to have an important role in nutrient transfers via their annual migration, and more locally, between the terrestrial and aquatic system due to their proximity to lake shores (Anderson, 2020). Geese populations in Greenland are changing dramatically with a decline in Greenland white‐fronted goose (*Anser albifrons flavirostris*) and increase in Canada goose (*Branta canadensis*) (Boyd & Fox, 2008; Fox & Glahder, 2010). Other studies have focussed on the role of goose faeces on soil nutrient pools adjacent to lakes (Bradley‐Cook et al., 2016; Côté et al., 2010; MacDonald et al., 2015; Mariash et al., 2018). Sjogersten et al. (2008) found that the C:N ratio in tundra ecosystems could be lowered due to external N inputs from geese that would increase the litter decomposition.

**FIGURE 1:**
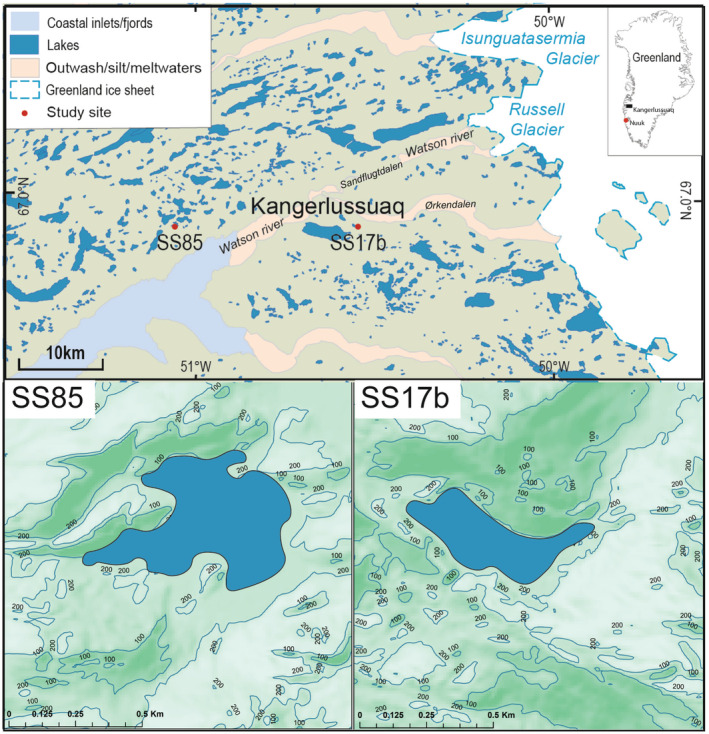
Location map of Kangerlussuaq in SW Greenland and insets showing the locations of the catchments in the study area

The area around Kangerlussuaq (Figure 1) is the largest ice‐free extent in western Greenland with a strong regional climate gradient, which influences both terrestrial vegetation and water chemistry (Anderson & Stedmon, 2007). As well as limnological surveys (Anderson et al., 2001; Osburn et al., 2019), there is a range of other ecological studies focused on vegetation phenology (Cahoon et al., 2016), C‐dynamics (Bradley‐Cook & Virginia, 2018; Ozols & Broll, 2003), and trophic interactions (Post & Høye, 2013). The area is particularly relevant in terms of arctic environmental change, because despite a cooling trend for much of the late‐20th century (Hanna et al., 2012), Kangerlussuaq is now warming quickly, with major changes in the timing of the onset of spring (Saros et al., 2019).

Recent soil‐focused work in the Kangerlussuaq area was reviewed by Anderson (2020), and highlighted the complex and heterogenous nature of soil types and cover in the Kangerlussuaq area, their relationship to local vegetation and hence, ultimately, regional microclimates. However, as well as the role of local landscape morphology (Henkner et al., 2016), there is also the possible impact of local wind erosion (Heindel et al., 2015), dust deposition (van Soest et al., 2022; Willemse et al., 2003), and impacts of grazers (Cahoon et al., 2012).

Given this background, this study focused on soil nutrient pools in small catchments in the Kangerlussuaq area of SW Greenland (Figure 1). The aim of the work was to compare nutrient pools in terms of topographic position and soil depth in low‐Arctic catchments, which were known to contrast in terms of muskox abundance and hence grazing effects. The introduction of muskox in 1962 offers a quasi‐natural experimental framework as they were introduced to the region in a clearly defined and spatially constrained area (Raundrup et al., 2012). As well as the assumed differences in the role of grazing, the project design used a classic soil “catena” approach (Milne, 1935), studying soil nutrient concentration on the convex ridge, slope and concave sites, as well as at different soil depths at each sample location. Specifically, we tested the following hypotheses: firstly, there were differences between soil C, N and P concentrations and their inter‐relationships across a soil catena, specifically between the ridge, slope, and valley of the catena; second, there were differences between soil C, N and P concentrations and their inter‐relationships between soil depth; and finally, there were differences in soil C, N and P concentrations and their inter‐relationships between the two catchments with contrasting muskox abundance (i.e., grazing history).

## 2 METHODS

### 2.1 Study area

The study area is located around the head of Kangerlussuaq (Søndre Strømfjord in Danish), a long fjord cut by outlet glaciers draining the Greenland ice sheet (Figure 1). Diverse gradients in climate and vegetation that function over a range of spatial and temporal scales encompass the area with distance away from the Greenland ice sheet. The present‐day ice margin is some 30 km to the east and there are now extensive outwash plains (sandurs), which are the source of surrounding loess deposits. The regional bedrock geology is predominantly ancient granodioritic gneiss with occasional ultra‐basic intrusions. There is only a thin scattering of glacial till on the higher ground. The local climate is low‐Arctic continental with a mean annual temperature of −6°C; summers are short but warm, temperatures can reach 16°C in early July.

The area is dry with annual precipitation <200 mm year^−1^ with most of the rainfall falling in the late summer. The low precipitation combined with warm, windy summers means that effective precipitation is negative (Cahoon et al., 2016). Snowfall is therefore critical for regional hydrology (and soil nutrient transfer) but much of the snow can sublimate in the late winter. Moisture stress is severe for terrestrial vegetation to the extent that south‐facing slopes are largely devoid of shrub tundra and have limited soil development. Permafrost is continuous but locally very spatially variable and the active layer is ~50 cm with maximum thaw depth reached in early August.

Following Bocher's (1949) initial research on soils in the Kangerlussuaq area, Henkner et al. (2016) classified soils in the Umimmalissuaq valley, about 30 km southeast of Kangerlussuaq, as Haplic Regosols and Cryosols. They, and a few others, have estimated the C content linked to vegetation, landscape position, or both (Bradley‐Cook & Virginia, 2018). The mineral soil C and N pools in the area differ substantially between graminoid and shrub dominated soils (Petrenko et al., 2016). Regionally, soils are thin, and deeper slightly acidic organic soils tend to be in the valleys and on the north facing slopes (Anderson, 2020).

The dry regional climate combined with strong katabatic winds flowing off the ice sheet, which deflate fine material on the sandurs, results in considerable dust deposition (van Soest et al., 2022). Extensive dust deposits can be observed on vegetation and snowbanks at the end of the winter and can form visible crusts locally, particularly where soils are thinner due to its incorporation into the organic matrix (Bocher, 1949). Historically, extreme phases of aeolian activity have formed recognisable layers in soil profiles providing depositional records (Willemse et al., 2003).

The terrestrial vegetation is classified as dwarf shrub tundra and is dominated by dwarf birch (*Betula nana*), grey willow (*Salix glauca*), crowberries (*Empetrum* sp.), Labrador tea (*Ledum* sp.), and blueberry (*Vaccinium* sp.); with grasses and cryptogams also common. The only large herbivore in the Kangerlussuaq area was the reindeer (*Rangifer tarandus*) until the introduction of muskox (*Ovibos moschatus*) into the area immediately south‐east of the airport around 1962 (Raundrup et al., 2012). Other terrestrial herbivores are arctic hare (*Lepus arcticus*) and geese. To date, anthropogenic influence on the environment is limited to long‐range atmospheric pollution (e.g. Bindler et al., 2001: see Anderson, 2020 for a discussion), but this may change if tourism increases or resource development occurs.

### 2.2 Site selection and sample collection

Samples were collected from two catchments (Figure 1) in August 2007, which were selected after consideration of regional environmental gradients and the known history of muskox introduction. Catchment codes refer to the numbering system used in limnological reports for this region (e.g. Anderson et al., 2001; Anderson & Stedmon, 2007). The numerical labels and prefix SS used in these studies have been retained here for consistency and allow data relating to the catchments from this study to be associated with previous work (e.g. Anderson et al., 2001; Saros et al., 2019). Based on basal radiocarbon dates of lake sediment cores, both catchments were deglaciated at a similar time, approximately 8500 cal yr BP (Bennike, 2000; McGowan et al., 2003).

**FIGURE 2:**
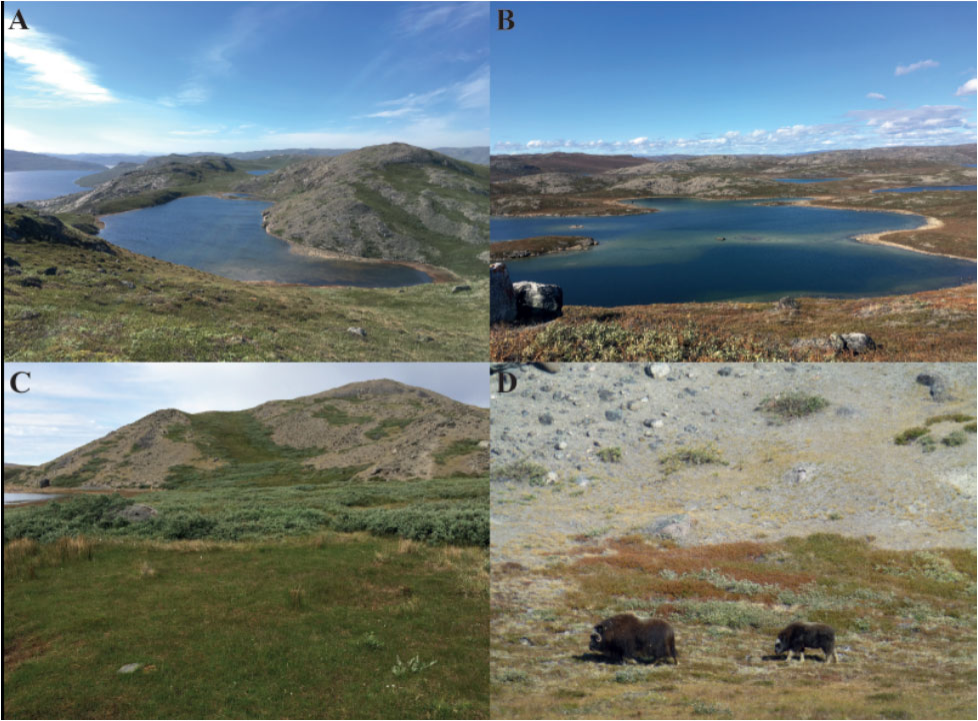
(a) Overview photo looking down from the north facing slope at SS17b; (b) overview photo looking down from the north facing slope at SS85 (note that this photo is taken later in the summer/Arctic autumn); (c) muskox “lawn” at SS17b, with the lake in the background. Note the contrast in vegetation; (d) a muskox with calf near the bare south facing slope at SS17b

Catchment 1 (hereafter SS17b) is located ~6 km south‐east of Kangerlussuaq airport, close to the oligosaline lake, Store Salt Sø (SS17), and the Sandflugtdalen‐Ørkendalen glacio‐fluvial system. The SS17b sample points were located on the north‐west facing slope, adjacent to a small freshwater lake (Figure 2a). This area has a substantial muskox population that grew from only 27 introduced individuals to an estimated population of 7–10,000 in the late 1990s and may currently be closer to 24,000 (Raundrup et al., 2012). The Watson River was a natural barrier to reindeer migration historically and they are rarely seen around SS17b, that is to the south of the river. Similarly, muskox were for many years restricted to the southern area but have now migrated and are common to the north of Sandflugtdalen. Vegetation is dominated by grassland with sparse low grey willow and dwarf birch shrubs (Figure 2a). The south facing slope and boulder areas are bare (Figure 2d). The damper, permafrost‐affected hummocks along the north facing slope are rich in mosses.

Catchment 2 (SS85) (Figure 2b) is located close to the head of the fjord, 20 km west of the airport. The sampling sites are on a north facing slope immediately north of the fjord, close to a small freshwater lake (SS85) which is close to a larger oligosaline lake, Braya Sø (SS4). South facing slopes are covered with grey willow, grasses but with extensive bare patches, whereas the north facing slopes are hummocky and dominated by dwarf shrub tundra. Muskox are very rare but there are occasional reindeer. Both catchments are impacted by dust deposition and investigations suggest that the rates are similar at both locations (van Soest et al., 2022).

### 2.3 Sampling protocol

Along the north facing slope of each catchment, a soil catena was identified and divided into three 10 m^2^ blocks at the ridge, slope, and valley bottom of each catena. Within these blocks, five sampling points were selected using random number generated coordinates. Samples were collected from soil litter, 0–5 cm depth below the litter layer, and 25–30 cm below the litter layer, resulting in a total of 90 samples across both sites. A 5‐cm long cylinder with a diameter of 4.8 cm was used and samples were deposited in sealable plastic bags and stored under refrigeration prior to analysis.

### 2.4 Sample analysis

The samples were dried at 105°C and analysed for bulk density, moisture content, total C (TC), N (TN), P (TP), δ^13^C and δ^15^N. Since there is no source of inorganic C in the study area (Henkner et al., 2016), the organic fraction is assumed to be equal to TC. A Carlo Erba NA 1500 element analyser (CE Instruments Ltc, Wigan, UK) linked with a Sercon 20:20 IRMS (Isotope Ratio Mass Spectrometer, Sercon Ltd., Crewe, UK) was used for the TC and TN content. δ ^13^C and δ^15^N were determined following the method described by Dixon et al. (2009). Wheat flour (IA R001, Iso‐Analytical, Crewe, UK) was used as a reference, and another wheat flour calibrated by Iso‐Analytical was used for quality control purposes with each batch of samples analysed. Total P in the litter and soil samples was obtained using the Saunders and Williams method (litter) or the Smith and Bain method (soil) and analysed colourimetrically as molybdenum blue using a spectrophotometer (Murphy & Riley, 1962).

Fresh soil samples were used for the quantification of the different forms of N and P. Samples for NH_4_^+^ (ammonium), NO_3_^−^ (nitrate) and NO_2_^−^ (nitrite) were extracted using 2 M KCl (Bremner & Keeney, 1966) and stored in a freezer at −14°C before analysis with a Bran + Luebbe segmented flow analyser (NO_3_^−^ + NO_2_^−^) (Hanley Technology, Perth, UK) or an Aquakem 250 discrete photometric analyser (NH_4_^−^) (Thermo Scientific, Hemel Hempstead, UK). Water soluble forms of P were analysed using the method of Turner and Haygarth (2001). An Aquakem 250 discrete photometric analyser was used to determine soil water extractable total P (WETP) (<0.45 μg) and soil water extractable reactive P (WERP) (<0.45 μg).

Two‐way anova was used to determine the significance of the difference for 14 parameters as given in the three hypotheses (there were differences between C, N and P concentrations and their interrelationships (1) across a catena, (2) with soil depth, and (3) between sites); statistical analyses were undertaken in Gentstat (10th edition). The TC, TN, and TP concentrations as g kg^−1^ were, for the ease of comparison to other studies, expressed as kg m^−2^ for the top 30 cm of the soil profile (Table 3). The following formula was used:

$$\mathrm{TC}=\left({\mathrm{BD}}^{*}1000\right)*C/\left(1,000,000\right)*t.$$

where *TC* is the total nutrient content in kg m^−2^, *BD* is the bulk density measured by dry weight of the sample / total volume of the sample (g cm^−3^), *C* is the nutrient concentration in mg kg^−1^, and *t* is the soil layer thickness (m). The average nutrient concentration (mg kg^−1^) of the 0–5 and 25–30 cm soil layers were averaged to calculate the content to 30 cm depth.

## 3 RESULTS

**FIGURE 3:**
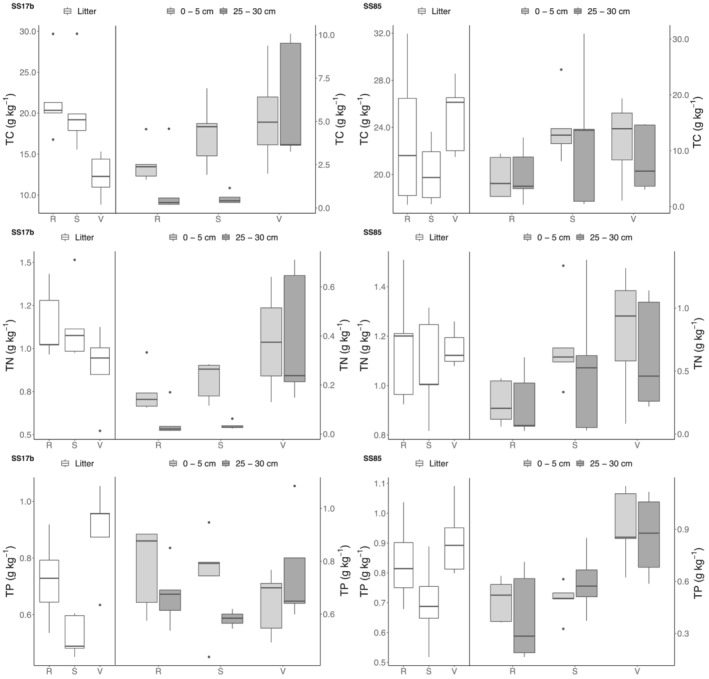
TC, TN and TP concentrations in g kg^−1^ separated by catchment (SS17b – SS85), catena position (R (ridge) – S (slope) – V (valley)) and sample depth (litter – 0–5 cm – 25–30 cm). Bold horizontal line indicates median, upper and lower limits of boxes indicate the 1st and 3rd quartiles, vertical lines indicate minimum and maximum values

Total C and TN concentrations (Figure 3) were on average lower at SS17b (7.88 g C kg^−1^ soil and 0.55 g N kg^−1^ in the valley; 8.43 g C kg^−1^ and 0.46 g N kg^−1^ on the slope; 8.46 g C kg^−1^ and 0.45 g N kg^−1^ on the ridge), compared to the concentrations at SS85 (15.1 g C kg^−1^ and 0.86 g N kg^−1^ at the valley sites; 15.4 g C kg^−1^ soil and 0.76 g N kg^−1^ on the slope; 11.3 g C kg^−1^ and 0.55 mg N kg^−1^ on the ridge). Total C was significantly different between site (*p* < 0.001), and sample depth (*p* < 0.001) although it only varied between catena positions for the surface sample (0–5 cm) (*p* < 0.05) (Tables 1 and 2). Concentrations decreased with soil depth and were on average highest in the valley positions, followed by the slopes and ridges. Total N was not significantly different for the litter layer. However, differences per soil depth were significant (*p* < 0.001) and so was the difference in concentration per catena position (at 0–5 cm) (*p* < 0.05) and site (*p* < 0.001) (Tables 1 and 2). The average TP was similar for both catchments with 0.70 g P kg^−1^ at SS17b and 0.69 g P kg^−1^at SS85. Overall, there was a significant difference in the TP between the two catchments (*p* < 0.001) (Tables 1 and 2). For TP in the litter, there was a significant difference between catena positions (*p* < 0.001) and a difference between catchments (*p* < 0.05) (Table 2). Total P concentrations in the litter layer were highest in the valley and lowest on the slopes (Figure 3).

**TABLE 1:** Summary of the statistical differences between the two catchments and three depths sampled (litter ‐ 0–5 cm ‐ 25–30 cm)

Parameter	Catchment	Depth	Catchment* depth
Bulk density	ns	***	Ns
Moisture content	***	***	***
TC	***	***	Ns
TN	***	***	Ns
TP	***	ns	Ns
C:N	***	ns	Ns
C:P	***	***	*
N:P	***	***	***
TON	***	***	***
NH_4_^+^	ns	*	ns
WETP	**	***	**
WERP	**	***	***
δ^13^C	ns	***	*
δ^15^N	*	***	ns

^*^ *p* < 0.05; ^**^ *p* < 0.01; ^***^ *p* < 0.001; ns = not significant.

Abbreviations: NH_4_^+^, ammonium; TC, total carbon; TN, total nitrogen; TON, total organic nitrogen; TP, total phosphorus; WERP, water extractable reactive phosphorus; WETP, water extractable total phosphorus.

**TABLE 2:** Summary of the statistical differences between the two catchments and three catena positions sampled

Parameter	Depth	Catchment	Position	Catchment*position
Bulk density	0–5 cm	ns	*	ns
	25–30 cm	ns	ns	ns
Moisture content	Litter	***	*	ns
	0–5 cm	***	*	ns
	25–30 cm	***	*	ns
TC	Litter	***	ns	***
	0–5 cm	***	*	ns
	25–30 cm	*	ns	ns
TN	Litter	ns	ns	ns
	0–5 cm	**	*	ns
	25–30 cm	*	ns	ns
TP	Litter	*	***	ns
	0–5 cm	ns	ns	***
	25–30 cm	ns	***	ns
C:N	Litter	***	ns	***
	0–5 cm	*	***	ns
	25–30 cm	ns	ns	ns
C:P	Litter	ns	***	*
	0–5 cm	***	**	*
	25–30 cm	***	ns	ns
N:P	Litter	ns	***	ns
	0–5 cm	***	*	*
	25–30 cm	***	ns	ns
TON	Litter	***	***	**
	0–5 cm	ns	ns	ns
	25–30 cm	ns	ns	ns
NH_4_^+^	Litter	ns	ns	ns
	0–5 cm	ns	ns	*
	25–30 cm	ns	*	ns
WETP	Litter	***	***	**
	0–5 cm	***	***	**
	25–30 cm	ns	ns	ns
WERP	Litter	***	***	**
	0–5 cm	ns	ns	ns
	25–30 cm	ns	ns	ns
δ^13^C	Litter	*	ns	ns
	0–5 cm	ns	ns	ns
	25–30 cm	ns	ns	ns
δ^15^N	Litter	*	***	***
	0–5 cm	ns	***	*
	25–30 cm	***	***	*

^*^ *p* < 0.05; ^**^ *p* < 0.01; ^***^ *p* < 0.001; ns = not significant.

Abbreviations: NH_4_^+^, ammonium; TC, total carbon; TN, total nitrogen; TON, total organic nitrogen; TP, total phosphorus; WERP, water extractable reactive phosphorus; WETP, water extractable total phosphorus.

**FIGURE 4:**
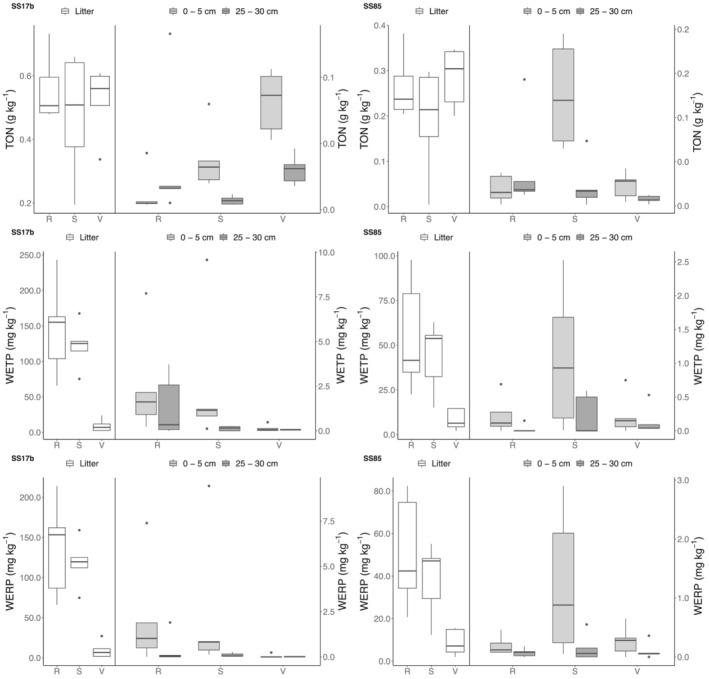
TON (g kg^−1^), WETP (mg kg^−1^) and WERP (mg kg^−1^) concentrations separated by catchment (SS17b ‐ SS85), catena position (R (ridge) ‐ S (slope) ‐ V (valley)) and sample depth (litter ‐ 0–5 cm ‐ 25–30 cm). Bold horizontal line indicates median, upper and lower limits of boxes indicate the 1st and 3rd quartiles, vertical lines indicate minimum and maximum values

The total organic N (TON) content of the litter layer was significantly (*p* < 0.001) higher at SS17b (~0.52 g N kg^−1^) compared to SS85 (~0.25 g N kg^−1^), but this difference between sites was reduced in the soil layers (Figure 4). Maximum surface layer TON occurred in the valley at SS17b (81.5 mg N kg^−1^), whereas the concentration was highest on the slope at SS85 (126 mg kg^−1^), and the pattern between catena positions was different for the 25–30 cm layer where both ridge positions had high TON. The average WETP across all sites was 22 mg kg^−1^, with 32 mg kg^−1^ at SS17b and a 12 mg kg^−1^ at SS85. For both catchments, the WERP values were only slightly smaller than the WETP values. There was a significant difference (*p* < 0.001) in WERP of the litter layer (Figure 4) between both catchments and catena positions. Soil WERP in the litter layers at both catchments was much less in the valleys compared to the slopes and ridges (9.51, 118, and 136 mg kg^−1^, respectively, at SS17b and 8.78, 38.5, and 50.9 mg kg^−1^, respectively, at SS85) (Figure 4). The WERP of the soil layers ranged between 0.01 and 2.33 mg kg^−1^ at SS17b and 0.07 and 1.23 mg kg^−1^ at SS85, substantially less than the litter layers. The water extractable total phosphorus (WETP) was significantly different (*p* < 0.001) in the soils of SS17b compared to SS85 (31.6 and 12.1 mg P kg^−1^ soil, respectively) (Tables 1 and 2). The WETP was significantly different with profile depth (*p* < 0.001), with litter layers at both sites having concentrations ca. 40–1000 higher than in the two soil horizons. However, there was only a significant effect of the slope position on WETP (*p* < 0.001) for the litter and the 0–5 cm soil layer. The mean WETP of the litter layer at the slope and ridge location at SS17b was 146 and 122 mg P kg^−1^, respectively, and more than double than at SS85 (55 and 44 mg P kg^−1^). WETP concentrations were smaller in litter layer at the valley positions with 9.5 mg P kg^−1^ at SS17b and 8.5 mg P kg^−1^ at SS85. A comparable site trend was apparent in both soil layers with <2.5 mg P kg^−1^ (Figure 4).

**FIGURE 5:**
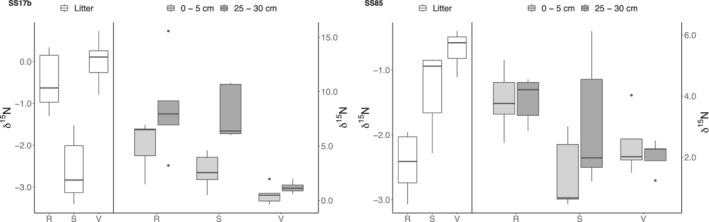
δ^15^N separated by catchment (SS17b ‐ SS85), catena position (R (ridge) ‐ S (slope) ‐ V (valley)), and sample depth (litter ‐ 0–5 cm ‐ 25–30 cm). Bold horizontal line indicates median, upper and lower limits of boxes indicate the 1st and 3rd quartiles, vertical lines indicate minimum and maximum values

There was a significant difference (*p* < 0.001) in δ^15^N (Figure 5) at 25–30 cm depth in the SS17b samples, where δ^15^N was 1.20‰ in the valley compared to 8.00‰ at the slope and 8.57‰ at the ridge. The difference was slightly less at the soil surface (range 0.53–5.12‰). Such variability was not observed at SS85 where δ^15^N was more uniform over soil depth in both the valley and ridge positions but with a slight increase with depth at the slope. The highest values were in general obtained on the ridges. The δ^15^N isotopic variation between the two catchments was significant (*p* < 0.05), with generally higher values at SS17b. δ^13^C was only significantly different with soil depth and did not vary substantially between catchments or catena positions (see Tables 1 and 2). However, δ ^13^C values increased significantly (*p* < 0.001) from −27.1 ‰ in the litter layer to −26.0 ‰ at 0–5 cm and −25.9 ‰ at 25–30 cm depth.

**FIGURE 6:**
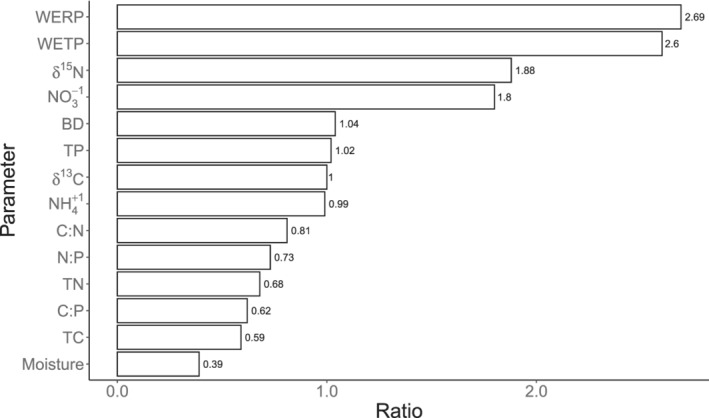
Ratios of mean parameters per catchment (SS17b divided by SS85). Parameter catchment‐based differences are significant *p* < 0.05, except BD, TP, δ^13^C and NH_4_^+^

To summarise the results: approximately, half of the measured parameters showed a significant difference between catchments (54%) and catena positions (48%), when specifically analysed by depth (Table 2). Catchment‐related differences were found in the litter (69%) and surface layer (57%) but less at the 25–30 cm soil depth (42%). For the catena positions, significant differences were mostly found in the 0–5 cm layer (69%) and the litter (58%), but again much less in the 25–30 cm layer (28%). The ratios of the mean parameters per catchment (SS17b divided by SS85) is given in Figure 6. These parameter catchment‐based differences are significant except for BD, TP, δ^13^C and NH_4_^+^. The overall differences between the catchments (with and without muskox) and soil depths (litter, 0–5 cm and 25–30 cm) were significant for 79% and 71%, respectively (Table 1).

## 4 DISCUSSION

The soil nutrient content at the two Kangerlussuaq study sites show clear spatial variability both at the landscape scale, and across the catena positions within each catchment. The results are discussed in terms of the broader spatial scale glacial landscape model proposed by Anderson (2007), as well as the more local scale catena approach (i.e. ridge‐slope‐valley comparison). The two catchments in the present study can be classified as distal in the terminology of Anderson (2007) given their distance from the present ice sheet and their similar age. The differences in soil nutrients between the two sites are also considered in terms of local‐to‐regional drivers, especially the effect of muskox grazing (Olofsson et al., 2001; Post & Høye, 2013), but also dust deposition (van Soest et al., 2022).

### 4.1 Differences among catena positions

Soils on slopes, as geomorphic process zones, are more vulnerable to erosion and are more heterogenous in curvature than, by definition, flat crests and valley positions (Miller & Schaetzl, 2016). Within a classically defined catena sequence, finer particles, that is, clays or OM, are transported from the upper slopes and redeposited in downslope positions (Rosenbloom et al., 2001). Higher nutrient content was found at the valley positions of both catchments (Figure 3). The increase in concentration from ridge to valley most likely relates to differences in soil moisture conditions and is less likely the result of erosional downslope transport of particulates and associated nutrients. This is because, as Pastick et al. (2017) argue, dry aerobic conditions lead to the absence of permafrost, higher rates of decomposition and mineralization, and thin surface organic layers in upland ecosystems. In contrast, flat low‐lying areas are typically underlain by near‐surface permafrost leading to perched water tables and anaerobic conditions that slow rates of microbial decomposition and the accumulation of large amounts of organic C.

### 4.2 Carbon

Tarnocai et al. (2009) estimated the C burden for the upper 0–100 cm in this area of Greenland to be 15.3 kg m^−2^ based on a small‐scale soil map and the average C content obtained from Inceptisols sampled within the northern circumpolar permafrost region. However, soil C content from the present study varied considerably at a local scale, both between catchments and catena positions, confirming the findings of Henkner et al. (2016), who worked closer to the ice sheet margin. The average TC content per catchment for the surface 30 cm was 13.68 kg C m^−2^ at SS17b and 34.08 kg m^−2^ at SS85 (Table 3). These values are higher than those found by Henkner et al. (2016) in the Umimmalissuaq valley, south of Kangerlussuaq (mean 9.9 kg C m^−2^, 8.35 kg C m^−2^ south‐facing and 11.45 kg C m^−2^ north‐facing) and 16.4 kg C m^−2^ (50 cm depth) reported by Bradley‐Cook and Virginia (2018).

**TABLE 3:** Nutrient stocks within the top 30 cm layers of the soil (kg m^−2^) per catena position and averaged over the entire catchment. The average nutrient concentration (mg kg^−1^) of the five replicates per catena position was used to calculate the nutrient content for the 0–5 and 25–30 cm layers, which were then extrapolated to 30 cm

		Ridge	Slope	Valley	Average
SS17b	TC	6.02 ± 0.06	8.27 ± 0.12	26.8 ± 0.22	13.7
	TN	0.36 ± 0.00	0.43 ± 0.00	1.83 ± 0.02	0.90
	TP	0.23 ± 0.01	0.23 ± 0.01	0.33 ± 0.01	0.26
SS85	TC	22.7 ± 0.26	37.9 ± 0.30	41.7 ± 0.50	34.1
	TN	1.01 ± 0.01	1.78 ± 0.02	2.94 ± 0.03	1.91
	TP	0.18 ± 0.01	0.16 ± 0.01	0.36 ± 0.01	0.24

Abbreviations: TC, total carbon; TN, total nitrogen; TP, total phosphorus.

There were no significant differences in δ^13^C between sites or location. The δ^13^C is on average −26‰ and comparable to observations across a range of lake‐catchments from near the ice sheet and westwards past Kangerlussauq airport to the head of the fjord (Osburn et al., 2019). As expected, δ^13^C increases with depth. In cold environments, soil bioturbation is extremely limited and organic matter decomposition, downward transfer, leaching and gaseous losses of isotopically lighter C all lead to increased δ^13^C with depth (Körner et al., 2016).

### 4.3 Nitrogen

The total N content was significantly different between the two sites, as well as among soil depth and catena positions (for the 0–5 cm layer only) (Tables 1 and 2). Bradley‐Cook and Virginia (2018) found that TN contents ranged from 0.06 to 4.85 kg N m^−2^ to a depth of 50 cm under varying vegetation types and estimated the average content to be 1.07 kg N m^−2^, results similar to those presented here: 0.90 kg N m^−2^ (SS17b) and 1.91 kg m^−2^ (SS85) (Table 3). In contrast to the TN content of the litter layer, TON was significantly different between the two sites (0.52 g N kg^−1^ at SS17b and 0.24 g N kg^−1^ at SS85) and substantially higher than the soil layers (0.03 g N kg^−1^ at SS17b and 0.04 g N kg^−1^ at SS85), which were not significantly different (Figure 4). Litter TON was higher at SS17b, whereas the soil TON content was greater at SS85.

The results show strong increases in the δ^15^N with soil depth (Figure 5). Plant δ^15^N is usually lower than that of soil organic matter (SOM), as reflected in the lower δ^15^N of the litter layers in both catchments, and increases as function of soil nitrogen cycling. In general, the fractionation sequence is δ^15^N_nitrate_ < δ^15^N_ammonium_ < δ^15^N_old organic matter,_ and the remaining non‐transformed SOM becomes enriched in δ^15^N with time, so deeper soil horizons commonly have more positive δ^15^N than topsoil (Robinson, 2001). The δ^13^C and δ^15^N values in litter and soils reflect the overall inputs, outputs, and transformations occurring and are gross indicators of the ecosystem C and N dynamics (Bol et al., 2005; Robinson, 2001; Wang et al., 2018). There is a global mechanistic link between δ^13^C and δ^15^N through the process of SOM decomposition and microbial processing and an imbalance in these isotopic values can indicate ecosystem sources of either soil C or N, inferring vegetation inputs (Nel et al., 2018). The greater δ^15^N difference between the litter and deeper soil (25–30 cm) observed in the valley and slopes at SS17b (9.5–10.5‰) and those at SS85 (4.5–6.5‰), suggest a more open, more labile N cycle (cf. Heindel et al., 2019).

### 4.4 Phosphorus

To date, published studies of soil P from west Greenland are rare. Canini et al. (2020) reported soil P in the range of ~0.06–0.40 g P kg^−1^ across three habitats in the area of Kobbefjord, Nuuk (ca. 64° N), but did not discuss these values further. The soil TP concentrations of SS17b and SS85 are higher with an overall mean of 0.69 (range 0.39–0.90) g P kg^−1^. The Kangerlussauq soils are similar to those from arable soils in the Netherlands (average 0.50 [range 0.16–0.92] g P kg^−1^; van der Wal et al., 2007) and grassland soils from England and Wales (averaged 0.94 [range 0.38–1.98] g P kg^−1^; Turner & Haygarth, 2003) even in the absence of fertiliser inputs. The source of the soil P in the Kangerlussuaq area reflects weathering from parent geological material (ancient granodioritic gneiss with ultra‐basic intrusions, glacial till) (Hawkings et al., 2016) and via aeolian deposition (van Soest et al., 2022).

As well as the input of P‐rich dust, the presence of muskox at SS17b elevated the labile WETP fractions in the soil surface litter fractions (Figure 4), clearly accelerating P cycling with inputs from dung and urine, with potential for further acceleration if vegetation greening increases. Because of the high P (i.e. total and labile water extractable “WE..” fractions), it can be postulated that the terrestrial ecosystems may, in future, become more N and temperature limited. The geochemical changes may result in increased production (greening) and altered vegetation composition, and potentially increased C decomposition and progression to less efficient systems that result in losses of nutrients (Wild et al., 2014).

### 4.5 Nutrient ratios

Nitrogen:phosphorus ratios ranged between 0.6 (SS17b slope 25–30 cm) and 22.1 (SS17b slope litter) in all samples. The average N:P at SS17b was 7.4 and 10.1 at SS85, which would imply that both sites are N‐limited based on Hobbie et al. (2002), who concluded that a N:P ratio > 16 indicates P limitation, while N:P < 14 is indicative of N limitation. However, in general, arctic ecosystems are particularly limited in N and P ions due to their dependence on the constraints of C mineralization (Schlesinger & Bernhardt, 2013).

Carbon:nitrogen ratios ranged between 13.7 (SS85 valley 25–30 cm) and 32.2 (SS85 ridge 25–30 cm) and the average C:N was 16.6 at SS17b and 20.5 at SS85. Lower C:N ratios at SS17b support the inference of grazing as a key process as the C:N ratio is negatively correlated with OM decomposition (Schlesinger & Bernhardt, 2013). The difference in N and δ^15^N between catchments is also likely caused by the presence of muskox at SS17b; δ^15^N is higher at the later compared to SS85. δ ^15^N is higher in grazed soils, indicating greater ecosystem N losses (Bradley‐Cook & Virginia, 2018). C:P ratios ranged between 10 (SS17b slope 25–30 cm) and 398 (SS17b slope litter) while the average C:P was 126 at SS17b and 202 at SS85. These ratios suggest that the vegetation at SS17b is less P‐limited than at SS85. Aanderud et al. (2018) found that an increase in soil P has a strong effect on the soil microbiological community and species composition, further affecting mineralization. Whether an increase in C mineralization directly increases N and P availability is doubtful (Aanderud et al., 2018).

### 4.6 Soil and vegetation differences between catchments

The spatial heterogeneity of the landcover in Greenland is linked to temperature and water balance (moisture) (Karami et al., 2018). The interaction between vegetation type and soil temperature is important in predicting ecosystem respiration, and although air temperature may not directly influence soil temperature at depth, it alters soil moisture content significantly (Cahoon et al., 2012). The altitudinal difference between SS17b and SS85 is small (ca. 30 m) but, together with aspect and overall relief, should be considered because of its influence on local air temperature and precipitation differences between these sites. These local differences in climate conditions can create variability in the start and length of the growing season at the local scale. A fivefold difference was detected in N mineralization among dry, moist and wet ecosystems in the foothills of Alaska's North Slope, using in situ soil incubations (Schlesinger & Bernhardt, 2013). These differences were largely due to variations in the quality of SOM and microclimate among ecosystems (Schlesinger & Bernhardt, 2013). SOM decomposition rates should be lower at SS17b (cold‐dry) compared to SS85 (warm‐moist) based on the microclimate and vegetation characteristics, but results indicate the opposite (Figure 6).

Soil mineralization rates are furthermore determined by the chemical and physical quality of plant litter. The vegetation cover in the Kangerlussuaq area is in general “greener” on north facing slopes. Grassland (steppe) becomes more prominent at higher altitude towards the ice margin, whereas the south facing slopes and areas further away from the ice sheet are dominated by dwarf shrub tundra. The SS85 catchment is mostly covered with mixed dwarf shrub vegetation but the south facing slopes are dominated by fell field or are bare depending on the curvature (Figure 2b). At SS17b, the northwest facing slope occupies the largest part of the catchment surrounding the lake and is mainly covered with dwarf shrub vegetation (Figure 2a). The south facing slope is a combination of bare, bedrock, and fell‐field while a steep and short north‐east facing slope is mainly covered with a mixture of vegetation types that gradually merges into fell‐field at the crest. Some steppe‐type vegetation was observed in the flat, slightly better drained parts of the area and at higher altitude (Figure 2c).

### 4.7 Muskox grazing and wider ecological factors

Recent change in arctic vegetation has focused on processes associated with climate change: warming, altered growing season length and soil biogeochemistry (e.g. Myers‐Smith et al., 2020; Treharne et al., 2019; Vonk et al., 2019). Altered precipitation leading to changed hydrological pathways and plant moisture stress are also important as are, in some areas, changed fire dynamics (Michaelides et al., 2019). Disturbance of tundra vegetation is potentially also caused by the increasing abundance and activity of animals (Ottersen et al., 2001). The populations of both reindeer and muskox have been shown to vary in relation to variable synoptic weather patterns in both east and west Greenland (notably the NAO; Post & Forchhammer, 2002). There is no evidence of forage competition between reindeer and muskox (Pedersen & Aastrup, 2000). However, given the rapid population increase in muskox numbers following their introduction in 1962 in a rather small geographic area around the SS17b catchment, it is reasonable to assume that they impacted vegetation and nutrient cycling at this site.

The tendency of muskox to form small clusters within clear geographic, territorial boundaries can lead to distinct “lawns” with contrasting vegetation composition, as observed at SS17b (Figure 2c). Apart from the nutrient input via dung, grazing and trampling also affect vegetation cover (van der Wal & Brooker, 2004). Due to both food preferences of the herbivores and different tolerance levels of plant species, these grazing‐related processes can affect soil temperature (Tomassini et al., 2019). Muskox are the largest terrestrial herbivores in the Arctic and their inter‐ and intra‐annual habitat selection are highly variable in relation to population density and snow cover (Olofsson et al., 2001). Patches of grassland were observed in flat, lower elevation parts of the catchment and gentle north facing slope at SS17b. These were also the locations where muskox were observed during several field campaigns (Figure 2d). Cahoon et al. (2012) demonstrated that muskox maintain a graminoid‐dominated ecosystem suppressing shrub abundance.

Overall, the TP content was different between the two catchments (Figure 6); however, this difference is mainly driven by the contrasting litter layers as the P content of the soil layers are similar (0.26 kg P m^−2^ at SS17b and 0.24 kg P m^−2^ at SS85). The variability within and between catchments suggests additional process complexity. Litter WERP and WETP are substantially greater at SS17b, and this can be related to the presence of muskox (a conclusion supported by δ^15^N). WERP is also the fraction of TP that is available for plant uptake and can therefore explain the variation in TP in the litter layer while there was no significant difference over the soil profile (cf. Haygarth et al., 2013).

Page et al. (2005) have shown that the WETP species are characteristically enriched in surface soils of grazed pasture. WERP was a high proportion of the WETP across most samples, and the residual is an approximate indicator of organic (unreactive) P in the soil solutions (cf. Turner & Haygarth, 2001). Here, organic P was estimated to be 25% in the soil solution across both catchments (31% in SS17b, 18% in SS85), a proportion relatively similar to that reported in leachate from intensively managed grassland in England (Turner & Haygarth, 2001). This is an interesting observation, but the cause and detailed mechanisms are impossible to discern from this study. Further investigations into the in‐soil nature and dynamics of the inorganic and organic P in the Kangerlussuaq area would be a priority recommendation for any future studies (George et al., 2018). Forman et al. (2007) suggest that in dry tundra ecosystems, where organic horizons are thin, P availability can be high in young soils with large pools of weatherable P in primary minerals, but low in more intensively weathered soils, especially if phosphates are tightly held to secondary minerals such as aluminium or iron oxides. This reaction of organic P with various soil minerals makes it difficult to estimate P mineralization rates.

The comparatively recent introduction (60 years) of muskox compared to the age of the catchments (ca. 8500 years) and, hence rates of soil development, suggests alternative mechanisms are required to explain the differences in P at depth. Although dust deposition exerts an influence on P dynamics, vegetation progressively shifts from N to P limitation with landscape and soil age (Anderson, 2007). Phosphorus cycling contrasts with N cycling as it is controlled by physical–chemical as well as biological processes; in the Kangerlussuaq area, this is primarily aeolian dust deposition (Reynolds et al., 2010).

The Greenland ice sheet is a hot spot of P production (Hawkings et al., 2016), and dust deposition provides an important linking mechanism of this nutrient to the terrestrial environment (Anderson et al., 2017). How much of this deposited P is biologically available is not known. The silt content and/or particle sizes of the soil samples are also unknown, which makes it difficult to make a distinction between these possible external P inputs. Although Hawkings et al. (2016) observed that both labile and refractory P contents increase with increasing silt content, indicating that dust inputs recently have supplied plant‐available P to the ecosystem.

While SS17b is located very close to the sandur (Figure 1), at both sites, the estimated dust deposition rates (as mineral matter) are similar (8.94 g m^−2^ year^−1^ at SS17b and 9.49 g m^−2^ year^−1^ at SS85 in 2018) (van Soest et al., 2022). In this context, it is unclear to what extent the two catchments responded differently to the effects of Little Ice Age (LIA) cooling, which may have re‐set landscape ontogeny and biogeochemical processes (Willemse et al., 2003). There would have been considerable die‐back of the vegetation at both catchments during the LIA. Wind‐scour and development of deflation hollows due to the cold katabatic winds may have been more pronounced at SS17b while it is clear that dust deposition was higher during the LIA (Anderson et al., 2012).

## 5 CONCLUSIONS AND SYNTHESIS

Significant differences were observed in C, N and P among catchments, catena positions, and with soil depth. Differences with soil depth and catena position are likely caused by soil–plant interactions and varying temperature and moisture content conditions related to topography. The presence of muskox contributes to differences in nutrient concentrations between catchments, although gradients in climate and vegetation with distance away from the ice sheet are also factors that cause differences in local processes between the two catchments. The effect of muskox on soil nutrients presented in this study are in line with the findings of Jørgenson et al. (2013), who showed the capability of muskox to control shrub growth on a landscape scale. The question remains whether soil nutrients pools are likely to be released in future under influence of climate change and whether the release of nutrients to the nearby lakes are going to be outcompeted by the increasing demands of terrestrial vegetation (Wookey et al., 2009). Because the soil P stores are high, there is a risk that this will accelerate the release of P from the soils to terrestrial and aquatic ecosystems, adding to the concerns about changing weathering rates across the Arctic (Hartmann et al., 2014). This underlines both the difficulties and the importance of an ecosystem approach in rapidly changing landscapes with multiple links and drivers. Moreover, it emphasises the importance of understanding permafrost‐affected soils and their role in global nutrient cycles.

## AUTHOR CONTRIBUTIONS

**Maud van Soest:** Visualization (lead); writing – original draft (lead); writing – review and editing (lead). **John Anderson:** Conceptualization (equal); writing – original draft (equal); writing – review and editing (equal). **Roland Bol:** Methodology (equal); writing – review and editing (supporting). **Liz Dixon:** Formal analysis (equal); methodology (equal). **Philip M Haygarth:** Conceptualization (lead); funding acquisition (lead); project administration (lead); writing – review and editing (supporting).

## Data Availability

The data that support the findings of this study are available from the corresponding author upon reasonable request.
